# A simple suturing technique to close arthroscopy portal wounds

**DOI:** 10.1308/003588412X13373405386015f

**Published:** 2012-09

**Authors:** M As-Sultany, P Porter

**Affiliations:** Yeovil District Hospital NHS Foundation Trust,UK

**Figure 1:**
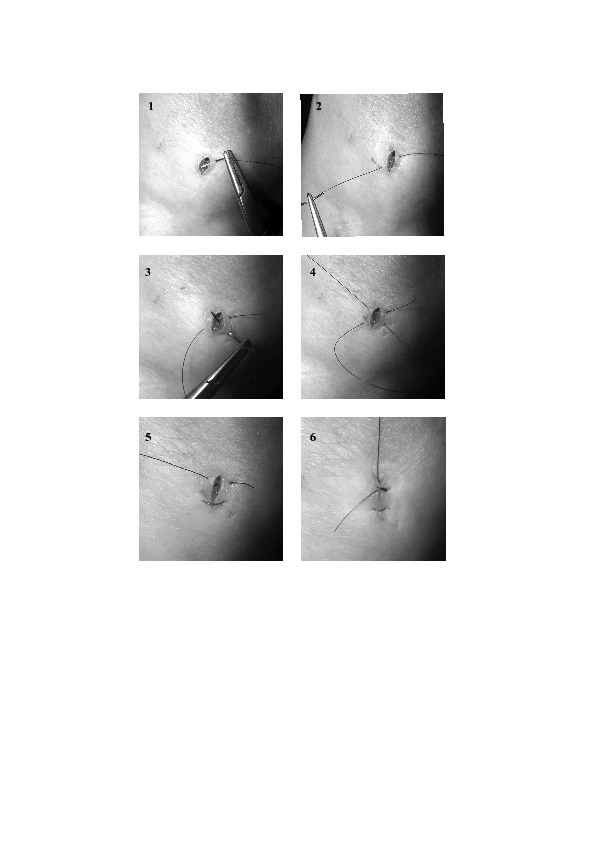
The figure-of-eight suture

We describe a simple and quick suturing technique that can be used to close arthroscopy portal wounds, resulting in excellent cosmetic appearance. A single suture is seamlessly run in the shape of a ‘figure of eight’ (Fig 1) to give neat apposition of the skin edges. Commonly used, single interrupted sutures result in a ‘dumbbell’ appearance of the wound while the mattress suture may cause rolling of the wound edges. We have found that this technique gives excellent cosmetic results with no wound complications.

